# Cutting back malaria: CRISPR/Cas9 genome editing of *Plasmodium*

**DOI:** 10.1093/bfgp/elz012

**Published:** 2019-07-29

**Authors:** Marcus C S Lee, Scott E Lindner, Jose-Juan Lopez-Rubio, Manuel Llinás

**Affiliations:** 1 Parasites and Microbes Programme, Wellcome Sanger Institute, Hinxton, UK; 2 Department of Biochemistry and Molecular Biology, Huck Center for Malaria Research, The Pennsylvania State University, Pennsylvania, USA; 3 Dynamique des Interactions Membranaires Normales et Pathologiques, UMR5235 CNRS, INSERM, Université Montpellier, Montpellier, France; 4 Department of Chemistry, The Pennsylvania State University, Pennsylvania, USA

**Keywords:** CRISPR/Cas9, malaria, genome editing, gene regulation, Plasmodium, parasite, apicomplexan

## Abstract

CRISPR/Cas9 approaches are revolutionizing our ability to perform functional genomics across a wide range of organisms, including the *Plasmodium* parasites that cause malaria. The ability to deliver single point mutations, epitope tags and gene deletions at increased speed and scale is enabling our understanding of the biology of these complex parasites, and pointing to potential new therapeutic targets. In this review, we describe some of the biological and technical considerations for designing CRISPR-based experiments, and discuss potential future developments that broaden the applications for CRISPR/Cas9 interrogation of the malaria parasite genome.

## Introduction

To functionally interrogate a genome requires the capability to delete, insert, rewrite and modify not just the specific nucleotides that compose the genome of an organism, but also to alter gene expression and the epigenetic marks that contribute to how the genome is used. Our ability to experimentally tinker with these aspects has been dramatically enabled in the past few years by the development of CRISPR/Cas9 approaches, which have transformed the speed and scale with which genome ‘editing’ can be achieved. This is true across fields, including parasitology and the study of apicomplexans that include *Plasmodium*, *Toxoplasma* and *Cryptosporidium*. In the latter case, CRISPR editing has been the key that has unlocked the ability to genetically manipulate *Cryptosporidium* in the lab [1], whereas the more genetically facile *Toxoplasma* has been taken to the next level of genome-scale exploration through forward genetic screens [2]. The *Plasmodium* parasite, on which this review is focused, lies somewhere in between these two extremes, with *in vitro* transfection methods in use for *Plasmodium falciparum* since 1995 and *in vivo* rodent malaria models with reasonable transfection efficiencies for genome scale approaches [3, 4]. Nonetheless, the introduction of CRISPR approaches has accelerated our collective ability to test essential pathways, generate conditional alleles, dissect domains and motifs and transplant single-nucleotide variants. In this review, we aim to highlight how CRISPR is being used in the malaria community, as well as synthesise our collective practical experience to date on how the system has worked, and the challenges for when it has not.

### A critical capability

Site-specific nucleases, including homing endonucleases (e.g. I-SceI), zinc-finger nucleases (ZFNs), Transcription activator-like effector nucleases (TALENS) and CRISPR/Cas9, share the ability to selectively trigger a double-strand break at a defined site in the genome. Genome engineering has simply exploited the desire of the cell to repair this adverse event. In many organisms, this repair can occur by the potentially error-prone pathway of non-homologous end joining, with the resulting indels generating a gene disruption [5]. *Plasmodium* species lack this ability, a double-edged sword that removes one facile method for generating gene disruptions (now used so effectively in *Toxoplasma*), but decreases the concern about potential off-target effects from cleavage at unintended loci. To our knowledge, off-target lesions have not been reported for *Plasmodium* after ZFN or CRISPR editing experiments, although a systematic examination of this question has yet to be performed. Furthermore, it should be noted that *Plasmodium* does possess the capability for microhomology-mediated end joining, which as the name suggests uses very short regions of homology flanking the double-strand break to repair the lesion, leading to potential indels. Nonetheless, this pathway appears to be relatively inefficient [6, 7], and more work needs to be done to understand how it might be exploited for targeted gene disruptions.

### CRISPR/Cas9 and its use in *Plasmodium*

CRISPR/Cas systems evolved in prokaryotes as a protective mechanism against invading bacteriophage and encode guide RNAs that program the Cas nuclease to bind and cut a specific target. Through evolution, prokaryotes have created a wide array of solutions to the same bacteriophage problem by inventing Cas proteins and guide RNAs of different compositions, structures and nucleic acid targets (reviewed in [8]). These nuclease-active Cas proteins have been effectively repurposed for gene editing applications (gene deletion, tag insertion and targeted mutations), whereas nuclease-dead variants (e.g. dCas9) have been used to affect gene regulation purposes (transcriptional activation or repression, epigenetic modification). While many Cas proteins have now been bioinformatically and experimentally defined, *Streptococcus pyogenes* Cas9 (SpCas9) is the most widely used, and to date all published work with *Plasmodium* parasites only uses SpCas9 and its variants. Given the restriction in *Plasmodium* species to homology-directed repair pathways, CRISPR/Cas9 is effectively a three-component system. Regardless of the specific approach, editing requires the delivery into the parasite of the Cas9 nuclease, the guide RNA(s) and a donor template for the cellular repair machinery to utilise. How each of these three components are delivered, and the design of the donor template to produce the intended modification, are discussed in more detail below.

### Species-specific CRISPR systems

The 1st reported CRISPR tools for *P. falciparum* were those by Ghorbal *et al.* [9] and Wagner *et al.* [10], which use a two-plasmid system to deliver Cas9, guide RNA (gRNA) and donor template. One critical feature for gRNA expression is the need for a precise transcription start at the 1st nucleotide of the gRNA (corresponding to the start of the target homology sequence), and thus in the majority of CRISPR systems, transcription has been driven from an RNA polymerase III promoter (although see below for alternate possibilities). The Ghorbal and Wagner studies solved this in two different ways, utilising respectively a parasite U6 snRNA promoter or a T7 phage promoter with corresponding co-expression of the T7 RNA polymerase. Variants of both the U6- and T7-based approaches have also been developed (e.g. [11, 12]), with one of our labs (Lee) exploiting a short U6 promoter to generate an all-in-one plasmid for delivering all three components that is suitable for relatively small (<1.5 kb) donors. The option of alternative positive selection (e.g. Blasticidin [13–15]) or negative selection markers, either on the donor plasmid [9] or the Cas9-gRNA plasmid [16], exists if counter-selection is desired. Additional methods to prevent the establishment of replicating episomes are to linearise the plasmid by restriction digest prior to transfection (as done by [9]) or to incorporate the specific gRNA site at the end of the donor sequence such that expression of the Cas9-gRNA within the parasite results in plasmid linearization (Lee, unpublished data).

In addition to *P. falciparum*, CRISPR reagents for another zoonotic malaria parasite, *Plasmodium knowlesi* have also been developed and are effective, which coupled with the higher transfection efficiency of this species should facilitate scaling of genetic modifications [17]. *Plasmodium knowlesi* CRISPR approaches can be used to functionally analyse genes and mechanisms relevant to the genetically intractable *Plasmodium vivax* parasite, to which *P. knowlesi* is closely related evolutionarily.

The rodent malaria species, *Plasmodium yoelii*, is also increasingly well resourced with CRISPR/Cas9 reagents. Jing Yuan and colleagues [18] developed a system that also uses RNA polymerase III (via a minimal U6 promoter) to transcribe gRNAs constitutively and at high levels. As only one drug-selectable marker, dihydrofolate reductase (DHFR), is commonly used in *P. yoelii* and the related *Plasmodium berghei* parasite, all of the necessary CRISPR DNA sequences were either packaged into a single plasmid, or separated across two plasmids (only one of which could be selected). Initial work demonstrated that gene editing by SpCas9 could be done efficiently in *P. yoelii*, but that the elimination of the plasmid was exceedingly challenging. The introduction of a negative drug selectable marker, the bifunctional yeast fusion cytosine deaminase/uracil phosphoribosyltransferase (yFCU) gene, allowed for the eventual elimination of parasites retaining the plasmid sequences and resulted in edited parasites that regained sensitivity to anti-folate drugs and could be edited again [19]. With this system, systematic interrogations of the ApiAP2 protein family and proteins related to ookinete motility have revealed key similarities and differences between *P. yoelii*, *P. berghei* and *P. falciparum* [19, 20]. Further developments in *P. yoelii* have included the production of a male/female reporter line expressing sex-enriched fluorescent proteins, as well as a parasite line constitutively expressing SpCas9 that would reduce the size of plasmids required [21, 22]. Recently, Walker and Lindner have reported the use of RNA polymerase II promoters to transcribe a ribozyme–guide–ribozyme system (CRISPR-RGR) in *P. yoelii* that can achieve high editing efficiencies for gene deletions and tag insertions [23]. This work also demonstrated that the number of gRNAs used influences gene editing outcomes, where using one gRNA can result in parasites bearing either plasmid integration and locus replacement gene edits, while using two gRNAs produces parasites with only locus replacement events. Moreover, this study also demonstrated that CRISPR interference (CRISPRi) is possible by placing nuclease dead variants of SpCas9 upstream of an endogenous gene. Because both of these systems from the Yuan and Lindner laboratories include DNA elements from both *P. yoelii* and *P. berghei* that have high sequence conservation, these plasmids should be functional in both species, although this remains to be tested. In addition, the development of CRISPR reagents specifically for *P. berghei* for gene deletion, editing and tagging is ongoing (B.Roberts and A.Waters, personal communication). A summary of CRISPR-based genome-editing experiments performed to date is provided in Table 1.

**Table 1:** Summary of genome-editing experiments performed in *Plasmodium* species

Genome-editing experiment	Target	Organism	Reference
Guide RNA database for *P. falciparum*	Multiple	*P. falciparum*	Ribeiro *et al.* [32]
Gene knockout or replacement	*kahrp*	*P. falciparum*	Ghorbal *et al*. [9]
	*kahrp, pfeba175*	*P. falciparum*	Wagner *et al*. [10]
	*pfvap1*	*P. falciparum*	Nacer *et al*. [67]
	*pfset2*	*P. falciparum*	Lu *et al*. [14]
	*pfptef*	*P. falciparum*	Chan *et al*. [68]
	*pfshelph2*	*P. falciparum*	Miliu *et al*. [69]
	*pycdpk3, pyctrp*	*P. yoelii*	Zhang *et al*. [19]
	*pyapiap2,* multiple	*P. yoelii*	Zhang *et al*. [20]
	*pfglo1 and pfcglo2*	*P. falciparum*	Wezena *et al*. [70]
	*pfcdpk2*	*P. falciparum*	Bansal *et al*. [71]
	*csp*	*P. falciparum*	Marin-Mogollon *et al*. [72]
	*pfp230p*	*P. falciparum*	Marin-Mogollon *et al*. [73]
	*pycdpk3, pyctrp*	*P. yoelii*	Qian *et al*. [21]
	*pfrh2a, pfrh2b*	*P. falciparum*	Campino *et al*. [74]
	*pfcdpk1*	*P. falciparum*	Bansal *et al*. [75]
	*pyalba4*	*P. yoelii*	Walker and Lindner [23]
	*pkdbpα* replacement with *pvdbp*	*P. knowlesi*	Mohring *et al.* [17]
Point mutation	*pfkelch13, pforc1*	*P. falciparum*	Ghorbal *et al*. [9]
	*pfcarl*	*P. falciparum*	LaMonte *et al*., [76]
	*pfugt, pfact*	*P. falciparum*	Lim *et al*. [11]
	*pfcdpk1*	*P. falciparum*	Bansal *et al*., [77]
	*pfmdr1*	*P. falciparum*	Ng *et al*., [78]
	*pfcpsf*	*P. falciparum*	Sonoiki *et al*., [79]
	*pfmdr1*	*P. falciparum*	Vanaerschot *et al*., [80]
	*pfatp4*	*P. falciparum*	Crawford *et al*. [26]
	*ul13*	*P. falciparum*	Wong *et al*., [81]
	*pfact1*	*P. falciparum*	Das *et al*., [82]
	*pfap2-i, pfmsp5* promoter	*P. falciparum*	Santos *et al*., [83]
	*pfdhodh*	*P. falciparum*	White *et al*., [84]
	*pfcoronin*	*P. falciparum*	Demas *et al*., [85]
	*pfatg18*	*P. falciparum*	Breglio *et al*., [86]
	*pfkelch13*	*P. falciparum*	Nair *et al*., [87]
	*pfkelch13*	*P. falciparum*	Payungwoung *et al*., [88]
Intron deletion	*var2csa*	*P. falciparum*	Bryant *et al*. [46]
Epitope tagging or conditional knockdown (DD, glmS or TetR-DOZI-binding aptamer)	*pfset7*	*P. falciparum*	Chen *et al*., [89]
	*plasmepsinix, plasmepsinx*	*P. falciparum*	Nasamu *et al*., [90]
	*pftric-Θ*	*P. falciparum*	Spillman *et al*. [12]
	*pfclpp, pfclpr*	*P. falciparum*	Florentin *et al*., [91]
	*pyp28*	*P. yoelii*	Zhang *et al*. [19]
	*pfck2β1, pfck2α, pfstk*	*P. falciparum*	Kuang *et al*. [15]
	*pfhsp70x*	*P. falciparum*	Cobb *et al*., [92]
	*pfgdv1*	*P. falciparum*	Filarsky *et al*., [93]
	*pfhsp70x*	*P. falciparum*	Kudyba *et al*. [42]
	*pfhsp101*	*P. falciparum*	Ho *et al*., [94]
	*pysep1*	*P. yoelii*	Qian *et al*. [21]
	*pfatg8*	*P. falciparum*	Walczak *et al*., [95]
	*pyccp2, pydhc1*	*P. yoelii*	Liu *et al*. [22]
	*pyalba4*	*P. yoelii*	Walker and Lindner [23]
	*pkama1, pkron2, pkmyoA, pkcrt, pkK13*	*P. knowlesi*	Mohring et al. [17]
Conditional KO (loxPintron)	DiCre driver lines	*P. falciparum*	Knuepfer *et al*. [16]
	*pfshelph2*	*P. falciparum*	Miliu *et al*., [69]
	*pfrhoph3*	*P. falciparum*	Sherling *et al*., [96]
Reporter lines	*gfp-luciferase*	*P. yoelii*	Lu *et al*. [14]
	*gfp* (*calmodulin, gapdh and hsp70* promoters)	*P. falciparum*	Mogollon *et al*. [13]
	*gfp*	*P. knowlesi*	Mohring *et al.* [17]

#### Purified Cas9 ribonucleoprotein

The majority of approaches described to date for *Plasmodium* species rely on delivery of the Cas9, gRNA and donor components on plasmids. Increasingly, however, CRISPR editing in mammalian systems is employing purified Cas9-gRNA ribonucleoprotein (RNP) that is complexed prior to delivery into the cell. In addition to potential increases in efficiency, the RNP approach does not consume any selectable markers and the short lifetime of the Cas9-gRNA RNP in the cell may limit off-target damage. Cas9 protein can be purchased from a number of commercial vendors, or expressed in bacteria [24]. Similarly, gRNAs can be generated by *in vitro* transcription from oligonucleotide templates (for example [25]) or commercially synthesised. To date, there has been only one report of using Cas9 RNP for editing in *Plasmodium*, which described the use of a Cas9-gRNA RNP co-electroporated with a 200-nucleotide single-stranded oligonucleotide as a donor to deliver a drug-resistance point mutation into the *pfatp4* gene [26]. Although parasites were recovered with the expected mutation, enrichment of the desired drug-resistant mutant required treatment of the culture with a PfATP4 inhibitor, reflecting the relative inefficiency of the editing event. Thus, the broad utility of the purified RNP method remains unclear, despite the potential advantages for streamlining of the design workflow.

**Figure 1:**
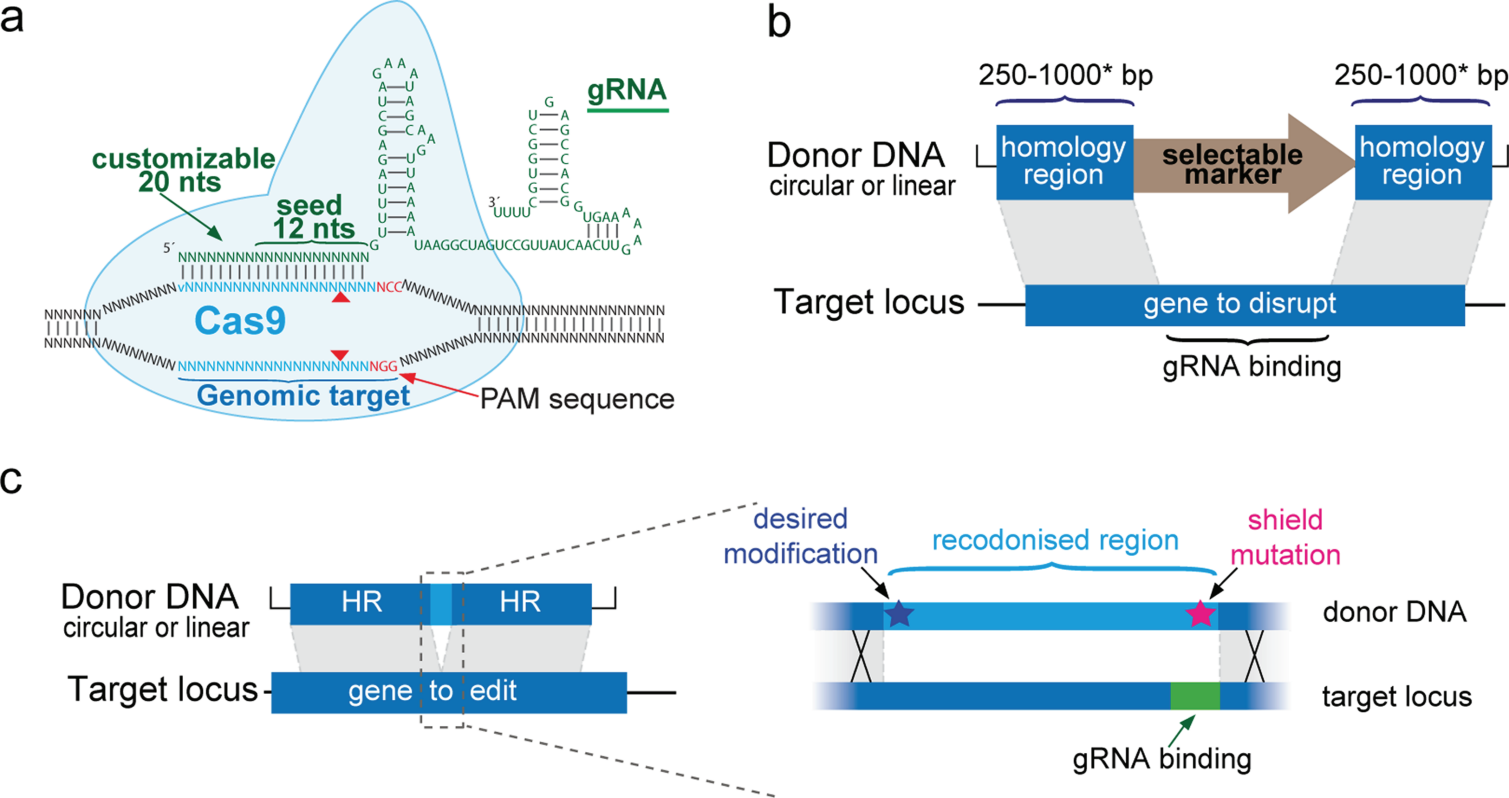
(a) Cas9 is directed to a specific genomic target by the first 20 nt of the gRNA, resulting in the generation of a double-strand break (red triangles). Donor design for (b) a typical gene-disruption experiment in *P. falciparum* and (c) marker-free genome editing of a point mutation. Silent ‘shield mutations’ prevent Cas9-gRNA cleavage of the edited locus. Additional silent mutations spanning the gap between the shield mutations and the desired modification can be introduced to help drive the repair event beyond the mutation-of-interest.

### Considerations for designing a CRISPR/Cas9 experiment

One of the positive features of the Cas9 system is that, unlike ZFNs and TALENs, the nuclease does not require modification to alter target specificity, greatly simplifying the design phase. Specificity instead is conferred by the 1st 20 nucleotides of the gRNA (Figure 1a). Nonetheless, careful selection of the appropriate gRNAs and consideration of donor template design can greatly increase the chances of a successful outcome. Below are several parameters that factor into experimental design.

#### Guide RNA design

The identification in the target genome of potential gRNAs that conform to a 20-nucleotide sequence followed by a (-NGG) protospacer adjacent motif (PAM) is most easily accomplished using one of the many freely available tools, and users may initially wish to evaluate the output from multiple sources. Some programs commonly used by our labs include Benchling (Biology Software, 2018), Protospacer [27], CHOPCHOP [28] and EuPaGDT [29], all of which score gRNAs on the basis of the number and position of mismatches at potential off-target sites in the genome. Notably, mutations in the ‘seed’ region (Figure 1a), the 12 nucleotides directly upstream of the PAM, are most disruptive to binding. This effect also factors into the choice of silent ‘shield’ mutations that are inserted into the donor template, described below.

In addition to the off-target score, some gRNA prediction tools provide an on-target score that aims to predict gRNA activity. The on-target score is modeled from a large-scale survey of the activity of several thousand gRNAs on a set of mammalian target genes [30, 31]. Whether this model is reflective of gRNA activity on more AT-rich genomes found in some *Plasmodium* species is not clear, and our collective experience to date indicates that even gRNAs with low on-target scores can be successfully used. Nonetheless, a recent study by Ribeiro *et al.* [32] suggests that on-target scores are predictive of success even in *P. falciparum*, and report an annotated list of all 662 795 potential gRNA sites in the *P. falciparum* genome. Another consequence of AT-rich genomes is the potential for poly-T stretches within the gRNA sequence, which could result in premature termination by RNA pol III and T7 RNAP, and thus should be avoided unless an RNA pol II expression system, like CRISPR-RGR, is used [23, 33]. Finally, recent ATAC-seq data on *P. falciparum* [34, 35] may be useful to inform gRNA selection to bias towards open chromatin or to troubleshoot unsuccessful editing events, as chromatin accessibility has been shown to affect editing in other systems [36].

A general rule of thumb for gRNA selection is to find guides that bind as close to the desired site of modification as possible, with the fewest predicted off-target effects [37]. In the case of editing a single point mutation, the gRNAs should ideally be located within 100–200 basepairs (bp) of the target site, with the frequency of capturing the desired mutation by the repair event decreasing with distance from the cut site. Similarly, insertion of tags or regulatory elements at the 5′ and 3′ ends of a gene constrain the choice of gRNAs available. For the creation of both point mutations and tag insertions, silent ‘shield’ mutations at the gRNA-binding site allow preservation of the coding sequence while preventing Cas9 cleavage of the donor plasmid or the correctly repaired genomic locus (Fig. 1c). Most disruptive to gRNA binding is mutation of the PAM, or if not possible, the introduction of mutations in the seed region. Gene deletions, on the other hand, afford the use of any gRNA within the deleted region (Figure 1b).

The selection of more than one gRNA per target is recommended to increase the odds of obtaining at least one active gRNA, with our labs typically selecting two gRNAs per editing event. In addition to the conventional use of these gRNAs individually, an alternate approach is to express multiple gRNAs in a single cell to improve efficiency and as a hedge against having one poorly active gRNA. A simple approximation of this approach is used by the Lee lab by co-transfecting two separate gRNA plasmids, relying on the propensity of *P. falciparum* to take up multiple plasmids during transfection (our unpublished data). However, a variety of more sophisticated methods for multiplex gRNA expression have been developed for mammalian cells, *Drosophila*, plants and other organisms. The approaches range from tiling multiple gRNA expression cassettes within a single plasmid, to the use of a single-polycistronic transcript that flanks each gRNA with tRNA [38] or ribozyme sequences [39], resulting in liberation by endogenous nucleases or self-cleavage, respectively. The advantage of the polycistronic approach is that the gRNAs are expressed from a single promoter, with promoter choice not restricted to RNA pol III-based expression, enabling stage-specific gRNA expression. Approaches for multiplex gRNA expression in *P. yoelii* using hammerhead and hepatitis delta virus ribozymes have now been developed (described above).

#### Donor design

The specifics of donor design are as diverse as the potential uses of CRISPR-gene disruption, single nucleotide modification, tagging or marker-free insertion of fluorescent reporters or conditional control elements. However, some general factors are relevant for all homology-directed repair approaches, with one primary consideration being the length of the homology region. In the absence of a nuclease-triggered double-strand break, long homology arms can assert a strong influence on the efficiency of gene targeting. For example, the *Plasmo*GEM large-scale knock-out project in *P. berghei* examined the efficiency of integration with homology arms ranging from 0.4 to 14 kb, and observed improved efficiency above 1.25 kb up to 10 kb [4]. In contrast, the majority of Cas9-based templates used in *Plasmodium* are at or below 1 kb, likely reflecting the stimulating effect of an induced double-strand break. Our collective experience to date indicates that homology regions of >250–1000 bp are sufficient (Figure 1b), a similar range also observed by Ribeiro *et al*. [32], with efficient editing of *P. yoelii* with homology arms as short 80–100 bp as reported by Walker and Lindner [23]. However, to date there have been no reports of success using the very short homology regions (<50 bp) that are effective in *Toxoplasma* [40, 41]. In a recent study, Kudyba *et al*. (2018) [42] tested insertion of a PCR-produced marker flanked by 50–100 bp homology regions, but saw little to no editing. To our knowledge, the smallest single-stranded oligonucleotide donor used successfully to date is the 200-nt repair template to introduce a drug-resistance mutation in PfATP4 [26]. However, it is unclear whether the relative inefficiency of this editing event is derived from the use of a short oligonucleotide template or the Cas9-RNP approach.

Another factor that influences donor design is the conversion tract length of the repair process from the site of the double-strand break, in other words, how far along the donor template that sequence changes are captured. This effect varies between organisms [43, 44], and at a practical level will inform how close the gRNA-binding site should be to the site of the desired mutation. Ideally, the double-strand break should be triggered directly at the site of the desired mutation, however in practice, this is often not possible. In *P. falciparum*, we have noted that when the desired point mutation or tag lies further than 100 bp from the shield mutations at the gRNA binding site, we observe variable capture (from 0–100%) of the desired event even if the shield mutations are edited with 100% efficiency. One solution is simply to perform replicate transfections in the hope that at least one will result in a conversion tract that covers the mutation of interest. However, an alternate strategy is to ‘recodonise’ the region between the gRNA site and the desired mutation, essentially disrupting the homology in the intervening space with silent mutations (Figure 1c). This stretch of silent mutations does not alter the protein coding sequence, but will ensure that the repair process is driven beyond the desired mutation before homology is encountered. Given the number of potential mutations to be introduced, the recodonising approach is best achieved using gene synthesis of the donor.

#### Challenges

A general caution for any genome-editing strategy should be the consideration of unexpected deletions and rearrangements that may be difficult to identify by standard PCR-based genotyping. Such events have been reported in mammalian systems [45], and the ability to perform whole-genome sequencing, including long-read sequencing, will be valuable in resolving these potential events. In addition, there are a number of *Plasmodium*-specific considerations that may impact editing outcomes. The *Plasmodium* genome contains a large number of multigene families, some with hundreds of members. If there is sufficient homology between family members, a potential challenge in targeting one specific member may be unintended repair from the paralogous gene sequences rather than the provided donor, as well as identifying unique gRNA sites in the first place. The latter point is addressed by the recent study of Ribeiro *et al*. [32], who annotate gRNAs that range in their ability to target a single family member, or that bind universally to all members.

Representatives of some of the largest multigene families, such as the *var*, *rifin* and *stevor* genes, are clustered in the subtelomeric regions, which presents an additional challenge for genome editing. When targeting the *var2csa* member of the *var* gene family, Bryant *et al*. [46] observed that the majority of editing events were not of the specific intron deletion that was desired, but rather the loss of the entire chromosome end downstream of the double-strand break to the telomere. This deleted region contained other non-essential genes and resulted in viable parasites that repaired the chromosome end with additional telomere repeats, in a process that is likely akin to telomere healing after spontaneous double-strand breaks [47]. Bryant *et al.* also noted that attempts to target other *var* gene members resulted in a similar outcome, suggesting that editing non-essential genes in subtelomeric regions will be challenging.

For more centrally located non-essential genes, an additional challenge arises when attempting to make non-disruptive edits such as point mutations and tag insertions. Rather than the desired edit, integration of the entire plasmid may occur in a manner similar to a conventional single-crossover recombination. This can result in apparent introduction of the silent mutations, and PCR genotyping of the 5′ and 3′ borders may appear correct; however, amplification across the locus may fail due to the insertion of the entire plasmid backbone. As this results in a gene disruption, it is not observed for essential genes; however, it appears to be a frequent competing outcome for non-essential genes, suggesting that extra care should be taken in genotyping these targets, with the isolation of clonal lines of particular importance (E.Hitz and T.Voss, personal communication, and [23]). Potential countermeasures could include making two cuts using two gRNAs, linearising the donor vector or the use of PCR or oligo donors, as well as negative selection on the plasmid backbone, although none of these measures are foolproof.

### CRISPR modulation of gene expression

The utility of the CRISPR system for interrogating the genome is not restricted to alteration of the nucleotide sequence, but for mammalian systems now extends to a dizzying array of potential options for regulating gene expression and modifying epigenetic marks. These alternate CRISPR activities rely on the DNA binding, but not cleaving, function of Cas9. By disabling the nuclease activity of Cas9, the resulting ‘dead’ Cas9 (or dCas9), when bound to the target gene, can interfere with RNA polymerase-mediated transcription [48]. Beyond simple steric interference, however, dCas9 has become a sophisticated platform for targeted delivery to genomic sites of a variety of add-on effector domains, either via direct fusion with dCas9, recruitment to a dCas9-linked epitope array such as the SunTag [49], or by using a modified gRNA that presents a protein-binding aptamer [50]. For a detailed review, see [51].

Enabling these methods for *Plasmodium* will require the development of parasite-specific tweaks to how these tools are deployed. For example, the strong viral transactivators such as VP64 that are routinely employed to increase transcription in mammalian systems (‘CRISPR activation’, ‘CRISPRa’) [52] were not thought to be able to function effectively in *Plasmodium* parasites. However, recent work from Lubin Jiang and colleagues demonstrated that a fusion of VP64 with the P65 and RTA transactivation domains can increase transcription of a targeted gene and affect related functions in that parasite [53]. Parasite transactivators, such as the Tati-2 [54] and TRAD4 domains [55], have been described, however these have yet to be validated in the dCas9 context, and more robust transactivators may ultimately be required. Transactivation domains are likely to be found in transcription factors, such as the ApiAP2 proteins, although effective transactivators remain elusive in *Plasmodium*. Effective stimulation of expression in mammalian systems requires the delivery of the transactivation domain near to the transcription start site [56], and the availability of genome-wide maps of transcription start sites for *P. falciparum* [57] will aid in this endeavour once effective systems are developed.

An alternate approach for gene regulation is to modify the epigenetic landscape through the recruitment of ‘writers’ and ‘erasers’ of epigenetic marks. Early examples of this approach in mammalian systems are the dCas9-mediated recruitment of the LSD1 histone demethylase to remove enhancer marks [58], and the core domain of the p300 histone acetyltransferase to deliver H3K27ac activation marks [59], with corresponding alterations in gene expression levels. Although the nature of the histone modifications in the parasite will differ from those employed in mammalian cells, our understanding of the types of marks, the proteins that deposit them and their regulatory consequences is increasing thanks to a number of genome-scale profiling studies [60–62]. Recently, the fusion of GCN5 or Sir2a to dCas9 was shown to activate or repress transcription of a target gene, respectively [53]. We anticipate that additional advances will continue to yield new CRISPR-mediated gene regulation approaches in the near future.

An additional challenge that may need to be overcome to improve CRISPR-mediated gene regulation of the AT-rich genome of *Plasmodium* species is the difficulty in identifying unique gRNA binding sites with the canonical (-NGG) PAM in intergenic regions, which have the highest AT-content of the genome. However, a wide variety of Cas9 variants with altered PAM specificities continue to be developed (see [63] for a comprehensive list), and new CRISPR nucleases are likely to emerge. One such nuclease that is well suited to AT-rich genomes is Cas12a (originally called Cpf1), which has a (TTTN-) PAM [64], and work by our groups is exploring whether variants of this nuclease (e.g. LbCas12a and AsCas12a) might provide a suitable system for *P. falciparum*, despite reported indiscriminate activity against single-stranded DNA [65]. Another intriguing possibility is development of RNA-targeting nucleases, such as Cas13, that may allow post-transcriptional regulation by RNA degradation as variants are developed that lack the non-specific RNAse activity (reviewed in [66]).

## Conclusions

CRISPR/Cas9 advances are accelerating the pace of *Plasmodium* research like never before. However, several important questions and challenges remain. First, it is not always possible to achieve complete editing of all parasites in the population of transfected parasites. What is the limiting event, and can it be overcome? We and others frequently observe that genome-editing outcomes from the same gRNA-donor pairing can be highly variable across multiple transfections. Looking ahead, the donor-free approaches of CRISPR-mediated transcriptional regulation will permit transfection of libraries of gRNAs for genetic screens. However, transfection efficiencies for some *Plasmodium* species remain low compared to other eukaryotes. The development of methods to improve transfection efficiency and reduce editing variability would be highly beneficial. Nonetheless, CRISPR approaches have heralded gains in the speed and efficiency of gene tagging and replacement, and are enabling precise genome modifications that are paving the way for testing active site mutations in enzymes and transcription factors (e.g. Hsp70X and AP2-I), the introduction of drug resistance alleles (e.g. *Kelch13*) and the dissection of redundant gene function. Few times in the brief history of molecular parasitology has such a modification to our ability to leapfrog forward been so great. We can now envision systematic whole-genome methods to knock out all non-essential genes and create reagents to query all essential genes. Once successful implementation of conditional gene knockdown systems based on dCas9 are viable, this will greatly expand our capacity to explore gene essentiality and the function of numerous unknown genes. We encourage the community to continue to develop and openly share these new tools for wide dissemination and adoption by anyone interested in molecular parasitology the world over.

## Key points

- CRISPR/Cas9 systems have now been developed for most experimentally-tractable *Plasmodium* species, expanding the range and precision of genome modification.
- A variety of approaches has been developed for delivery of the key components: the Cas9 nuclease, the guide RNA and the donor. Donor homology length and guide RNA selection are among the key considerations for experimental design.
- Challenges for experimental design include targets located in subtelomeric regions and the AT-rich genomes of some *Plasmodium* species.

## Notes

**Marcus Lee** is a group leader in the Parasites and Microbes Programme at the Wellcome Sanger Institute.

## Notes

**Scott E. Lindner** is an assistant professor of Biochemistry and Molecular Biology, and co-director of the Huck Center for Malaria Research at The Pennsylvania State University.

## Notes

**Jose-Juan Lopez-Rubio** is a group leader in the Laboratory Dynamique des Interactions Membranaires Normales et Pathologiques at the University of Montpellier.

## Notes

**Manuel Llinás** is a professor of biochemistry and molecular biology, professor of chemistry and co-director of the Huck Center for Malaria Research at The Pennsylvania State University.

## References

[1] Vinayak S, Pawlowic MC, Sateriale A, et al. Genetic modification of the diarrhoeal pathogen Cryptosporidium parvum. Nature 2015;523:477–80. 26176919 10.1038/nature14651 PMC4640681

[2] Sidik SM, Huet D, Ganesan SM, et al. A genome-wide CRISPR screen in toxoplasma identifies essential apicomplexan genes. Cell 2016;166:1423–35.e12. 27594426 10.1016/j.cell.2016.08.019 PMC5017925

[3] Wu Y, Sifri CD, Lei HH, et al. Transfection of *Plasmodium falciparum* within human red blood cells. Proc Natl Acad Sci U S A 1995;92:973–77. 7862676 10.1073/pnas.92.4.973 PMC42619

[4] Bushell E, Gomes AR, Sanderson T, et al. Functional profiling of a *Plasmodium* genome reveals an abundance of essential genes. Cell 2017;170:260–72.e8. 28708996 10.1016/j.cell.2017.06.030 PMC5509546

[5] Lee AH, Symington LS, Fidock DA DNA repair mechanisms and their biological roles in the malaria parasite *Plasmodium falciparum*. Microbiol Mol Biol Rev 2014;78:469–86. 25184562 10.1128/MMBR.00059-13 PMC4187680

[6] Kirkman LA, Lawrence EA, Deitsch KW Malaria parasites utilize both homologous recombination and alternative end joining pathways to maintain genome integrity. Nucleic Acids Res 2014;42:370–79. 24089143 10.1093/nar/gkt881 PMC3874194

[7] Singer M, Marshall J, Heiss K, et al. Zinc finger nuclease-based double-strand breaks attenuate malaria parasites and reveal rare microhomology-mediated end joining. Genome Biol 2015;16:49. 26573820 10.1186/s13059-015-0811-1 PMC4647826

[8] Knott GJ, Doudna JA CRISPR-Cas guides the future of genetic engineering. Science 2018;361:866–69. 30166482 10.1126/science.aat5011 PMC6455913

[9] Ghorbal M, Gorman M, Macpherson CR, et al. Genome editing in the human malaria parasite *Plasmodium falciparum* using the CRISPR-Cas9 system. Nat Biotechnol 2014;32:819–21. 24880488 10.1038/nbt.2925

[10] Wagner JC, Platt RJ, Goldfless SJ, et al. Efficient CRISPR-Cas9-mediated genome editing in *Plasmodium falciparum*. Nat Methods 2014;11:915–18. 25108687 10.1038/nmeth.3063 PMC4199390

[11] Lim MY, LaMonte G, Lee MC, et al. UDP-galactose and acetyl-CoA transporters as *Plasmodium* multidrug resistance genes. Nat Microbiol 2016;1:16166. 27642791 10.1038/nmicrobiol.2016.166 PMC5575994

[12] Spillman NJ, Beck JR, Ganesan SM, et al. The chaperonin TRiC forms an oligomeric complex in the malaria parasite cytosol. Cell Microbiol 2017;19. 10.1111/cmi.12719 PMC5429184 28067475

[13] Mogollon CM, van Pul FJ, Imai T, et al. Rapid generation of marker-free *P. falciparum* fluorescent reporter lines using modified CRISPR/Cas9 constructs and selection protocol. PLoS One 2016;11:e0168362. 27997583 10.1371/journal.pone.0168362 PMC5172577

[14] Lu J, Tong Y, Pan J, et al. A redesigned CRISPR/Cas9 system for marker-free genome editing in *Plasmodium falciparum*. Parasit Vectors 2016;9:198. 27066899 10.1186/s13071-016-1487-4 PMC4828878

[15] Kuang D, Qiao J, Li Z, et al. Tagging to endogenous genes of *Plasmodium falciparum* using CRISPR/Cas9. Parasit Vectors 2017;10:595. 29197418 10.1186/s13071-017-2539-0 PMC5712073

[16] Knuepfer E, Napiorkowska M, van Ooij C, Holder AA Generating conditional gene knockouts in plasmodium - a toolkit to produce stable DiCre recombinase-expressing parasite lines using CRISPR/Cas9. Sci Rep 2017;7:3881. 28634346 10.1038/s41598-017-03984-3 PMC5478596

[17] Mohring F, Hart MN, Rawlinson TA, et al. Rapid and iterative genome editing in the zoonotic malaria parasite *Plasmodium knowlesi*: provides new tools for *P. vivax* research *Elife*, 2019;8:e45829. 31205002 10.7554/eLife.45829 PMC6579517

[18] Zhang C, Xiao B, Jiang Y, et al. Efficient editing of malaria parasite genome using the CRISPR/Cas9 system. *MBio* 2014;5:e01414. 24987097 10.1128/mBio.01414-14 PMC4161241

[19] Zhang C, Gao H, Yang Z, et al. CRISPR/Cas9 mediated sequential editing of genes critical for ookinete motility in *Plasmodium yoelii*. Mol Biochem Parasitol 2017;212:1–8. 28034675 10.1016/j.molbiopara.2016.12.010 PMC5580835

[20] Zhang C, Li Z, Cui H, et al. Systematic CRISPR-Cas9-mediated modifications of *Plasmodium yoelii* ApiAP2 genes reveal functional insights into parasite development. MBio 2017;8(6):e01986–17. 29233900 10.1128/mBio.01986-17 PMC5727417

[21] Qian P, Wang X, Yang Z, et al. A Cas9 transgenic plasmodium yoelii parasite for efficient gene editing. Mol Biochem Parasitol 2018;222:21–28. 29684399 10.1016/j.molbiopara.2018.04.003 PMC11002757

[22] Liu C, Li Z, Jiang Y, et al. Generation of *Plasmodium yoelii* malaria parasite carrying double fluorescence reporters in gametocytes. Mol Biochem Parasitol 2018;224:9555–66. 10.1016/j.molbiopara.2018.07.010 30040976

[23] Walker MP, Lindner SE Ribozyme-mediated, multiplex CRISPR gene editing and CRISPRi in *Plasmodium yoelii J Biol Chem*. 2019;294:9555–66. 10.1074/jbc.RA118.007121 PMC6579477 31043479

[24] Lingeman E, Jeans C, Corn JE Production of purified CasRNPs for efficacious genome editing. Curr Protoc Mol Biol 2017;120:31.10.1–31.10.19. 10.1002/cpmb.43 28967993

[25] Lin S, Staahl BT, Alla RK, Doudna JA Enhanced homology-directed human genome engineering by controlled timing of CRISPR/Cas9 delivery. Elife 2014;3:e04766. 25497837 10.7554/eLife.04766 PMC4383097

[26] Crawford ED, Quan J, Horst JA, et al. Plasmid-free CRISPR/Cas9 genome editing in *Plasmodium falciparum* confirms mutations conferring resistance to the dihydroisoquinolone clinical candidate SJ733. PLoS One 2017;12:e0178163. 28542423 10.1371/journal.pone.0178163 PMC5439709

[27] MacPherson CR, Scherf A Flexible guide-RNA design for CRISPR applications using Protospacer Workbench. Nat Biotechnol 2015;33:805–6. 26121414 10.1038/nbt.3291

[28] Montague TG, Cruz JM, Gagnon JA, et al. CHOPCHOP: a CRISPR/Cas9 and TALEN web tool for genome editing. Nucleic Acids Res 2014;42:W401–7. 24861617 10.1093/nar/gku410 PMC4086086

[29] Peng D, Tarleton R EuPaGDT: a web tool tailored to design CRISPR guide RNAs for eukaryotic pathogens. Microb Genom 2015;1:e000033. 28348817 10.1099/mgen.0.000033 PMC5320623

[30] Doench JG, Fusi N, Sullender M, et al. Optimized sgRNA design to maximize activity and minimize off-target effects of CRISPR-Cas9. Nat Biotechnol 2016;34:184–91. 26780180 10.1038/nbt.3437 PMC4744125

[31] Doench JG, Hartenian E, Graham DB, et al. Rational design of highly active sgRNAs for CRISPR-Cas9-mediated gene inactivation. Nat Biotechnol 2014;32:1262–67. 25184501 10.1038/nbt.3026 PMC4262738

[32] Ribeiro JM, Garriga M, Potchen N, et al. Guide RNA selection for CRISPR-Cas9 transfections in *Plasmodium falciparum*. Int J Parasitol 2018;48:825–32. 29906414 10.1016/j.ijpara.2018.03.009 PMC9093057

[33] Ray M, Lee YW, Hardie J, et al. CRISPRed macrophages for cell-based cancer immunotherapy. Bioconjug Chem 2018;29:445–50. 29298051 10.1021/acs.bioconjchem.7b00768 PMC6063311

[34] Toenhake CG, Fraschka SA, Vijayabaskar MS, et al. Chromatin accessibility-based characterization of the gene regulatory network underlying *Plasmodium falciparum* blood-stage development. Cell Host Microbe 2018;23:557–69.e9. 29649445 10.1016/j.chom.2018.03.007 PMC5899830

[35] Ruiz JL, Tena J, Bancells C, et al. Characterization of the accessible genome in the human malaria parasite *Plasmodium falciparum*. Nucleic Acids Res 2018;46:9414–31. 30016465 10.1093/nar/gky643 PMC6182165

[36] Wu X, Scott DA, Kriz AJ, et al. Genome-wide binding of the CRISPR endonuclease Cas9 in mammalian cells. Nat Biotechnol 2014;32:670–76. 24752079 10.1038/nbt.2889 PMC4145672

[37] Paquet D, Kwart D, Chen A, et al. Efficient introduction of specific homozygous and heterozygous mutations using CRISPR/Cas9. Nature 2016;533:125–29. 27120160 10.1038/nature17664

[38] Port F, Bullock SL Augmenting CRISPR applications in drosophila with tRNA-flanked sgRNAs. Nat Methods 2016;13:852–54. 27595403 10.1038/nmeth.3972 PMC5215823

[39] Gao Y, Zhao Y Self-processing of ribozyme-flanked RNAs into guide RNAs in vitro and in vivo for CRISPR-mediated genome editing. J Integr Plant Biol 2014;56:343–49. 24373158 10.1111/jipb.12152

[40] Shen B, Brown KM, Lee TD, Sibley LD Efficient gene disruption in diverse strains of toxoplasma gondii using CRISPR/CAS9. MBio 2014;5:e01114. 24825012 10.1128/mBio.01114-14 PMC4030483

[41] Sidik SM, Hackett CG, Tran F, et al. Efficient genome engineering of *Toxoplasma gondii* using CRISPR/Cas9. PLoS One 2014;9:e100450. 24971596 10.1371/journal.pone.0100450 PMC4074098

[42] Kudyba HM, Cobb DW, Florentin A, et al. CRISPR/Cas9 gene editing to make conditional mutants of human malaria parasite *P. falciparum*. J Vis Exp 2018;139. 10.3791/57747 PMC6235188 30295650

[43] Carroll D, Beumer KJ Genome engineering with TALENs and ZFNs: repair pathways and donor design. Methods 2014;69:137–41. 24704173 10.1016/j.ymeth.2014.03.026 PMC4175112

[44] Elliott B, Richardson C, Winderbaum J, et al. Gene conversion tracts from double-strand break repair in mammalian cells. Mol Cell Biol 1998;18:93–101. 9418857 10.1128/mcb.18.1.93 PMC121458

[45] Kosicki M, Tomberg K, Bradley A Repair of double-strand breaks induced by CRISPR-Cas9 leads to large deletions and complex rearrangements. Nat Biotechnol 2018;36:765–71. 30010673 10.1038/nbt.4192 PMC6390938

[46] Bryant JM, Regnault C, Scheidig-Benatar C, et al. CRISPR/Cas9 genome editing reveals that the intron is not essential for var2csa gene activation or silencing in *Plasmodium falciparum*. MBio 2017;8:4,e00729–17. 10.1128/mBio.00729-17 PMC5513710 28698275

[47] Calhoun SF, Reed J, Alexander N, et al. Chromosome end repair and genome stability in *Plasmodium falciparum*. MBio 2017;8,4:e00547–17. 28790200 10.1128/mBio.00547-17 PMC5550746

[48] Qi LS, Larson MH, Gilbert LA, et al. Repurposing CRISPR as an RNA-guided platform for sequence-specific control of gene expression. Cell 2013;152:1173–83. 23452860 10.1016/j.cell.2013.02.022 PMC3664290

[49] Tanenbaum ME, Gilbert LA, Qi LS, et al. A protein-tagging system for signal amplification in gene expression and fluorescence imaging. Cell 2014;159:635–46. 25307933 10.1016/j.cell.2014.09.039 PMC4252608

[50] Konermann S, Brigham MD, Trevino AE, et al. Genome-scale transcriptional activation by an engineered CRISPR-Cas9 complex. Nature 2015;517:583–88. 25494202 10.1038/nature14136 PMC4420636

[51] Dominguez AA, Lim WA, Qi LS Beyond editing: repurposing CRISPR-Cas9 for precision genome regulation and interrogation. Nat Rev Mol Cell Biol 2016;17:5–15. 26670017 10.1038/nrm.2015.2 PMC4922510

[52] Mali P, Aach J, Stranges PB, et al. CAS9 transcriptional activators for target specificity screening and paired nickases for cooperative genome engineering. Nat Biotechnol 2013;31:833–38. 23907171 10.1038/nbt.2675 PMC3818127

[53] Xiao B, Yin S, Hu Y, et al. Epigenetic editing by CRISPR/dCas9 in *Plasmodium falciparum*. Proc Natl Acad Sci U S A 2019;116:255–60. 30584102 10.1073/pnas.1813542116 PMC6320497

[54] Meissner M, Krejany E, Gilson PR, et al. Tetracycline analogue-regulated transgene expression in *Plasmodium falciparum* blood stages using toxoplasma gondii transactivators. Proc Natl Acad Sci U S A 2005;102:2980–85. 15710888 10.1073/pnas.0500112102 PMC548799

[55] Pino P, Sebastian S, Kim EA, et al. A tetracycline-repressible transactivator system to study essential genes in malaria parasites. Cell Host Microbe 2012;12:824–34. 23245327 10.1016/j.chom.2012.10.016 PMC3712325

[56] Gilbert LA, Horlbeck MA, Adamson B, et al. Genome-scale CRISPR-mediated control of gene repression and activation. Cell 2014;159:647–61. 25307932 10.1016/j.cell.2014.09.029 PMC4253859

[57] Adjalley SH, Chabbert CD, Klaus B, et al. Landscape and dynamics of transcription initiation in the malaria parasite *Plasmodium falciparum*. Cell Rep 2016;14:2463–75. 26947071 10.1016/j.celrep.2016.02.025 PMC4806524

[58] Kearns NA, Pham H, Tabak B, et al. Functional annotation of native enhancers with a Cas9-histone demethylase fusion. Nat Methods 2015;12:401–3. 25775043 10.1038/nmeth.3325 PMC4414811

[59] Hilton IB, D’Ippolito AM, Vockley CM, et al. Epigenome editing by a CRISPR-Cas9-based acetyltransferase activates genes from promoters and enhancers. Nat Biotechnol 2015;33:510–17. 25849900 10.1038/nbt.3199 PMC4430400

[60] Hoeijmakers WA, Stunnenberg HG, Bártfai R Placing the *Plasmodium falciparum* epigenome on the map. Trends Parasitol 2012;28:486–95. 22999479 10.1016/j.pt.2012.08.006

[61] Duffy MF, Selvarajah SA, Josling GA, Petter M Epigenetic regulation of the *Plasmodium falciparum* genome. Brief Funct Genomics 2014;13:203–16. 24326119 10.1093/bfgp/elt047

[62] Gupta AP, Bozdech Z Epigenetic landscapes underlining global patterns of gene expression in the human malaria parasite, *Plasmodium falciparum*. Int J Parasitol 2017;47:399–407. 28414071 10.1016/j.ijpara.2016.10.008

[63] Adli M The CRISPR tool kit for genome editing and beyond. Nat Commun 2018;9:1911. 29765029 10.1038/s41467-018-04252-2 PMC5953931

[64] Zetsche B, Gootenberg JS, Abudayyeh OO, et al. Cpf1 is a single RNA-guided endonuclease of a class 2 CRISPR-Cas system. Cell 2015;163:759–71. 26422227 10.1016/j.cell.2015.09.038 PMC4638220

[65] Chen JS, Ma E, Harrington LB, et al. CRISPR-Cas12a target binding unleashes indiscriminate single-stranded DNase activity. Science 2018;360:436–39. 29449511 10.1126/science.aar6245 PMC6628903

[66] Terns MP CRISPR-based technologies: impact of RNA-targeting systems. Mol Cell 2018;72:404–12. 30388409 10.1016/j.molcel.2018.09.018 PMC6239212

[67] Nacer A, Claes A, Roberts A, et al. Discovery of a novel and conserved *Plasmodium falciparum* exported protein that is important for adhesion of PfEMP1 at the surface of infected erythrocytes; Cell. Microbiol 2015;17:1205–16. 25703704 10.1111/cmi.12430 PMC4654329

[68] Chan S, Frasch A, Mandava CS, et al. Regulation of PfEMP1-VAR2CSA translation by a *Plasmodium* translation-enhancing factor; Nat Microbiol 2017;2:17068. 28481333 10.1038/nmicrobiol.2017.68

[69] Miliu A, Lebrun M, Braun-Breton C, et al. Shelph2, a bacterial-like phosphatase of the malaria parasite *Plasmodium falciparum*, is dispensable during asexual blood stage. PLoS ONE 2017;12:e01087073. 10.1371/journal.pone.0187073 PMC5658161 29073264

[70] Wezena CA, Alisch R, Golzmann A, et al. The cytosolic glyoxalases of *Plasmodium falciparum* are dispensable during asexual blood-stage development. Microb Cell 2017;5:32–41. 29354648 10.15698/mic2018.01.608 PMC5772037

[71] Bansal A, Molina-Cruz A, Brzostowski J, et al. *Plasmodium falciparum* Calcium-Dependent Protein Kinase 2 Is Critical for Male Gametocyte Exflagellation but Not Essential for Asexual Proliferation. MBio 2017;8:8–397. 10.1128/mBio.01656-17 PMC5646254 29042501

[72] Marin-Mogollon C, van Pul FJA, Miyazaki S, et al. Chimeric *Plasmodium falciparum* parasites expressing *Plasmodium vivax* circumsporozoite protein fail to produce salivary gland sporozoites. Malar. J. 2018;17:288. 30092798 10.1186/s12936-018-2431-1 PMC6085629

[73] Marin-Mogollon C, van de Vegte-Bolmer M, van Gemert G-J, et al. The *Plasmodium falciparum* male gametocyte protein P230p, a paralog of P230, is vital for ookinete formation and mosquito transmission. Sci Rep 2018;8:14902. 30297725 10.1038/s41598-018-33236-x PMC6175877

[74] Campino S, Martin-Menedez A, Kemp A, et al. A forward genetic screen reveals a primary role for *Plasmodium falciparum* Reticulocyte Binding Protein Homologue 2a and 2b in determining alternative erythrocyte invasion pathways. PLoS Pathog 2018;14:e1007436. 30496294 10.1371/journal.ppat.1007436 PMC6289454

[75] Bansal A, Molina-Cruz A, Brzostowski J, et al. PfCDPK1 is critical for malaria parasite gametogenesis and mosquito infection. Proc. Natl. Acad. Sci. U.S.A. 2018;115:774–9. 29311293 10.1073/pnas.1715443115 PMC5789930

[76] LaMonte G, Lim MY-X, Wree M, et al. Mutations in the *Plasmodium falciparum* Cyclic Amine Resistance Locus (PfCARL) Confer Multidrug Resistance. MBio 2016;7:455. 10.1128/mBio.00696-16 PMC4958248 27381290

[77] Bansal A, Ojo KK, Mu J, et al. Reduced Activity of Mutant Calcium-Dependent Protein Kinase 1 Is Compensated in *Plasmodium falciparum* through the Action of Protein Kinase G. MBio 2016;7:1629. 10.1128/mBio.02011-16 PMC5142624 27923926

[78] Ng CL, Siciliano G, Lee MCS, et al. CRISPR-Cas9-modified pfmdr1 protects *Plasmodium falciparum* asexual blood stages and gametocytes against a class of piperazine-containing compounds but potentiates artemisinin-based combination therapy partner drugs. Mol. Microbiol. 2016;101:381–93. 27073104 10.1111/mmi.13397 PMC4958522

[79] Sonoiki E, Ng CL, Lee MCS, et al. A potent antimalarial benzoxaborole targets a *Plasmodium falciparum* cleavage and polyadenylation specificity factor homologue. Nat Commun 2017;8:14574. 28262680 10.1038/ncomms14574 PMC5343452

[80] Vanaerschot M, Lucantoni L, Li T, et al. Hexahydroquinolines are antimalarial candidates with potent blood-stage and transmission-blocking activity. Nat Microbiol 2017;2:1403–14. 28808258 10.1038/s41564-017-0007-4 PMC5708124

[81] Wong W, Bai X-C, Sleebs BE, et al. Mefloquine targets the *Plasmodium falciparum* 80S ribosome to inhibit protein synthesis. Nat Microbiol 2017;2:17031. 28288098 10.1038/nmicrobiol.2017.31 PMC5439513

[82] Das S, Lemgruber L, Tay CL, et al. Multiple essential functions of *Plasmodium falciparum* actin-1 during malaria blood-stage development. BMC Biol. 2017;15:70. 28810863 10.1186/s12915-017-0406-2 PMC5557482

[83] Santos JM, Josling G, Ross P, et al. Red Blood Cell Invasion by the Malaria Parasite Is Coordinated by the PfAP2-I Transcription Factor. Cell Host Microbe 2017;21:731–41.e10. 28618269 10.1016/j.chom.2017.05.006 PMC5855115

[84] White J, Dhingra SK, Deng X, et al. Identification and Mechanistic Understanding of Dihydroorotate Dehydrogenase Point Mutations in *Plasmodium falciparum* that Confer in Vitro Resistance to the Clinical Candidate DSM265. ACS Infect Dis 2019;5:90–101. 30375858 10.1021/acsinfecdis.8b00211 PMC6467762

[85] Demas AR, Sharma AI, Wong W, et al. Mutations in *Plasmodium falciparum* actin-binding protein coronin confer reduced artemisinin susceptibility. Proc. Natl. Acad. Sci. U.S.A. 2018;115:12799–12804. 30420498 10.1073/pnas.1812317115 PMC6294886

[86] Breglio KF, Amato R, Eastman R, et al. A single nucleotide polymorphism in the *Plasmodium falciparum* atg18 gene associates with artemisinin resistance and confers enhanced parasite survival under nutrient deprivation. Malar. J. 2018;17:391. 30367653 10.1186/s12936-018-2532-x PMC6204056

[87] Nair S, Li X, Arya GA, et al. Fitness Costs and the Rapid Spread of kelch13-C580Y Substitutions Conferring Artemisinin Resistance. Antimicrob. Agents Chemother 2018;62:1025. 10.1128/AAC.00605-18 PMC6125530 29914963

[88] Payungwoung T, Shinzawa N, Hino A, et al. CRISPR/Cas9 system in *Plasmodium falciparum* using the centromere plasmid. Parasitol 2018;67:605–8. 10.1016/j.parint.2018.06.002 29886342

[89] Chen PB, Ding S, Zanghì G, et al. *Plasmodium falciparum* PfSET7: enzymatic characterization and cellular localization of a novel protein methyltransferase in sporozoite, liver and erythrocytic stage parasites. Sci Rep 2016;6:21802. 26902486 10.1038/srep21802 PMC4763181

[90] Nasamu AS, Glushakova S, Russo I, et al. Plasmepsins IX and X are essential and druggable mediators of malaria parasite egress and invasion. Science 2017;358:518–22. 29074774 10.1126/science.aan1478 PMC5928414

[91] Florentin A, Cobb DW, Fishburn JD, et al. PfClpC Is an Essential Clp Chaperone Required for Plastid Integrity and Clp Protease Stability in *Plasmodium falciparum*. Cell Rep 2017;21:1746–56. 29141210 10.1016/j.celrep.2017.10.081 PMC5726808

[92] Cobb DW, Florentin A, Fierro MA, et al. The Exported Chaperone PfHsp70x Is Dispensable for the *Plasmodium falciparum* Intraerythrocytic Life Cycle. mSphere 2017;2:610. 10.1128/mSphere.00363-17 PMC5615134 28959740

[93] Filarsky M, Fraschka SA, Niederwieser I, et al. GDV1 induces sexual commitment of malaria parasites by antagonizing HP1-dependent gene silencing. Science 2018;359:1259–63. 29590075 10.1126/science.aan6042 PMC6219702

[94] Ho C-M, Beck JR, Lai M, et al. Malaria parasite translocon structure and mechanism of effector export. Nature 2018;561:70–75. 30150771 10.1038/s41586-018-0469-4 PMC6555636

[95] Walczak M, Ganesan SM, Niles JC, et al. ATG8 Is Essential Specifically for an Autophagy-Independent Function in Apicoplast Biogenesis in Blood-Stage Malaria Parasites. MBio 2018;9:749. 10.1128/mBio.02021-17 PMC5750400 29295911

[96] Sherling ES, Knuepfer E, Brzostowski JA, et al. The *Plasmodium falciparum* rhoptry protein RhopH3 plays essential roles in host cell invasion and nutrient uptake. Elife 2017;6:D539. 10.7554/eLife.23239 PMC5365315 28252384

